# Positive reputation for altruism toward future generations regardless of the cost for current others

**DOI:** 10.3389/fpsyg.2022.895619

**Published:** 2023-01-24

**Authors:** Yukako Inoue, Nobuhiro Mifune, Tatsuyoshi Saijo

**Affiliations:** ^1^Research Institute for Future Design, Kochi University of Technology, Kochi, Japan; ^2^School of Economics and Management, Kochi University of Technology, Kochi, Japan; ^3^Research Institute for Humanity and Nature, Kyoto, Japan

**Keywords:** altruism, future generations, intergenerational dilemma, sustainability, reputation, evaluation

## Abstract

Recently, altruism toward future generations (future altruism) has become a hot research topic. Although future altruism has been observed in several previous experiments, it is not yet clear when and why people are more likely to engage in future altruism. Drawing upon the empirical literature of reputation and cooperation, we predicted that future altruism brings reputational disadvantages. Accordingly, we investigated whether future altruism was evaluated positively or negatively by others in the current generation in two vignette studies (total *N* = 1,237). Contrary to our initial prediction, we found that future altruism was positively evaluated even when it decreased the payoff of the members of the current generation. The difference in the evaluation of future altruism, as opposed to unsustainable current-generation focused behavior, was most pronounced when people do not know how a future altruist allocates rewards among individuals in the current generation. However, the positive evaluation of future altruism did not stem from the expectation that future altruists would also be altruistic toward the current generation. These results indicated that reputational benefits (i.e., positive reputation from others in the current generation) promote future altruism.

## 1. Introduction

Recently, intergenerational problems, such as climate change and biodiversity loss, have attracted greater attention. It has become urgent to address these problems in the course of developing a sustainable society (United Nations, 2015). Intergenerational problems often entail the intergenerational dilemma, namely, conflict of interest between current and future generations (Wade-Benzoni and Tost, 2009); if we pursue the efficiency and benefit of our current generation, future generations will bear the cost, lose benefits, and/or face survival challenges. A growing body of research on altruism toward future generations (future altruism) has shown that people can behave altruistically toward the future, at least, in certain situations (Hauser et al., 2014; Saijo, 2019; Lohse and Waichman, 2020), and identified several psychological factors underlying future altruism (Wade-Benzoni, 2008; Wade-Benzoni, 2019 for review; Wade-Benzoni et al., 2008; Wade-Benzoni and Tost, 2009; Tost et al., 2015; Bang et al., 2017). However, the degree to which people display future altruism depends on the situation, and it is unknown in what situation people can behave altruistically toward future generations. In this study, we investigated whether reputational disadvantage can be a boundary condition.

Altruistic behavior toward future generations is particularly challenging as compared to that toward the current generation. When individuals interact with others in the same generation, the interaction can continue, and they can correspondingly expect that their altruistic behavior can be reciprocated (Trivers, 1971). Contrastingly, however, people cannot have such an expectation when their altruistic behavior is directed toward future generations; obviously, others in future generations cannot time-travel to reciprocate the altruism that others in the past displayed (Wade-Benzoni and Tost, 2009). Given that people cannot expect to receive a return favor, it may be reasonable that rational individuals pursue their personal interests and benefits for their own generation.

Given that future altruism is theoretically challenging, it is of vital importance to empirically examine whether and to what extent people actually exhibit altruistic behavior toward future generations. To address this, researchers have developed new experimental economic games. The two most common games are the intergenerational goods game (IGG; Hauser et al., 2014) and the intergenerational sustainable dilemma game (ISDG; Kamijo et al., 2017). In these two games, groups of participants represent different generations, and they sequentially make decisions. It is important that, in these games, a group affects the size of public goods (i.e., benefits) of subsequent groups (i.e., generation) but not vice versa. If individuals prefer to pursue their interests, this maximizes their benefit but is detrimental to future groups. Contrastingly, if they make a sustainable decision, they do not maximize the benefit for their generation but preserve the size of public goods for future groups. In sum, these two games share two key features of the intergenerational dilemma: the unidirectionality of resource flow and the conflict of interest between the current and future generations.

Previous studies have shown that individuals display more altruistic behavior toward future generations in the IGG than in the ISDG. In the IGG, approximately 70% of the participants consistently left sufficient resources to subsequent generations (Hauser et al., 2014; Lohse and Waichman, 2020; Klaser et al., 2021). In the ISDG, by contrast, the proportion of groups that chose the sustainable option was only approximately 30% in general (Kamijo et al., 2017; Shahrier et al., 2017). We argue that game structure may play a pivotal role in the observed difference in future altruism between the two games.

In the IGG, each participant independently decides how much resource they would like to take from the common resource pool. If the remainder of the common resource pool falls below a threshold, the next group receives nothing. In this game, therefore, future altruism costs each participant but one’s decision to benefit the future generation does not negatively influence the payoffs of others in the same generation. In the ISDG, players in the same group collectively choose between sustainable and unsustainable options (intergenerational decision). The sustainable option brings less benefits to the current generation than the unsustainable option, but the unsustainable option reduces the size of the public goods for the next group. In this game, thus, one’s decision to preserve the public goods for the next group (i.e., future altruism) lowers the benefit for their group. Future altruism in the IGG only costs oneself, but that in the ISDG costs their group as a whole. We argue that the difference in the game structure helps us understand why people display more future altruism in the IGG than in the ISDG (see also Böhm et al., 2020).

Why do people exhibit less future altruism when it costs their group than when it only costs themselves? We postulate that it can be because people assign different reputations to future altruism costing a group and that costing only oneself. Reputation creates incentives to behave altruistically toward strangers (Wu et al., 2016). Altruistic people are evaluated positively by third parties, and they can receive various benefits, including receiving altruistic behavior from others (indirect reciprocity; Nowak and Sigmund, 1998, 2005; Wedekind and Milinski, 2000; Milinski et al., 2001), building long-term relationships (Barclay, 2010), and improving their status (Hardy and Van Vugt, 2006; Van Vugt and Hardy, 2010). Conversely, people who behave exploitatively and selfishly toward others are evaluated negatively and are de facto excluded from the cooperative relationship (Feinberg et al., 2014; see also Halevy et al., 2012). When future altruism costs others in the same generation, those who display future altruism may earn a negative reputation and this may explain why individuals in the ISDG are much more reluctant to behave altruistically toward future generations compared to those in the IGG.

However, the role of reputation in the ISDG is not that simple; two diametrically opposite predictions can be made as to how people evaluate future altruism that costs the current generation as a whole. On the one hand, as we predicted in the previous paragraph, people may assign a bad reputation to future altruists who reduce the benefits for the current generation (Feinberg et al., 2014) in exchange for benefits for future groups. This brings a double disadvantage to future altruists; they not only fail to maximize their own payoff but also fail to build cooperative relationships with members of the current generation owing to their bad reputation. In this case, reputation may become an obstacle to future altruism. Conversely, people may positively evaluate future altruism despite that it reduces benefits for the current generation, if they can focus on the benefits that future altruists leave for future generations. As mentioned above, people tend to positively evaluate those who behave altruistically toward others in general (Hardy and Van Vugt, 2006; Barclay, 2010), and this may hold even when recipients of altruistic behavior are those in future generations. In this case, reputation may promote future altruism. Thus, while it seems reasonable to expect that the decreased future altruism in the ISDG is driven by reputational concern, this prediction deserves careful consideration, and it is of vital importance to first elucidate how individuals perceive future altruism that costs the current generation.

In the current research, therefore, we investigated whether future altruists in the ISDG, in which future altruism reduces the benefits of the current generation, would earn a negative or a positive reputation. In addition, we manipulated the amount of costs that future altruism makes members of the current generation incur. It is assumed that the fewer costs they have to incur, the more positive reputation future altruists earn. To address this, in the present research, we let future altruists decide the division of the money among members of the current generation; in one condition, the future altruist selfishly allocated money and imposed most of the costs associated with future altruism on the other member. In another condition, they equally allocated money and split the costs equally. In the last condition, they altruistically allocated money and bore most of the costs. This manipulation helped us elucidate whether the evaluation of future altruism depended on its cost for members of the current generation.

In Study 1, we exploratorily examined the evaluation of future altruists and unsustainable persons in the ISDG, manipulating the division of money among members of the current generation. In Study 2, we confirmed whether the results of Study 1 could be replicated with preregistration, preregistering the experimental method, statistical method, and hypotheses. In addition, we examined whether the estimation of the money allocation among the current generation had a mediating effect on the evaluation when actual money allocation was unknown. These studies revealed when future altruists can earn good reputations and suggest how reputation systems in the current generation work for future altruism.

## 2. Study 1

### 2.1. Method

#### 2.1.1. Participants

The survey form was prepared in Japanese and participants were recruited through an online research company (Cross Marketing Inc., Japan). We requested this company to equally assign participants’ gender and age in each of the four between-participants conditions. The number of participants who successfully answered the comprehensive check questions and completed the ISDG evaluation task was predetermined at 1,000 (500 men and 500 women; 125 men and 125 women in each condition; *M_*age*_* = 45.15, *SD* = 14.77). Because this was the first study on evaluating future altruism, the effect size was unpredictable. Therefore, we tentatively set the sample size at 250 in each condition.

We obtained ethics approval from the ethics committee of Kochi University of Technology, which met the requirements of the Declaration of Helsinki. We obtained informed consent from all participants; the description of this survey was displayed on the first page, and participants started the survey only after they agreed to participate.

#### 2.1.2. Procedure

After giving consent, participants answered demographic questions (sex, age, and residential area). Participants then read the instructions of the ISDG and answered comprehension check questions about the ISDG to ensure that they correctly understood the structure of the game (see Supplementary material for more details). Participants who correctly answered at least two out of three comprehension check questions proceeded to the next part. They were presented with the correct answers and explanations of the comprehension check questions and they started the ISDG evaluation task (see ISDG Evaluation task section). Finally, they answered some questions about personality (see Section 5 of the Supplementary material). Participants who failed to correctly answer at least two of the comprehension check questions were excluded from the study at this point and dismissed.

#### 2.1.3. ISDG evaluation task

This vignette task was developed to measure the evaluation of intergenerational behaviors in the ISDG, in which we manipulated the intergenerational altruism and the amount of costs that the current generation has to incur (i.e., intragenerational decision-making). Each group in the ISDG, which represented one generation, consisted of two people with different roles: the decision-maker (DM) and the evaluator. The DM made two decisions in the ISDG. In the first decision (the intergenerational decision), the DM chose between unsustainable option A and sustainable option B. The payoffs of the intergenerational decision are represented in Table 1. For instance, if the DM chooses option B, the first generation gets 1800 yen, which is 600 yen less than it would earn if the DM chose option A. While the sustainable option leads to less earning for the first generation than the unsustainable one, it is more beneficial for the subsequent generations. If the DM in the first generation chooses unsustainable option A, the sustainable and unsustainable options in the second generation yield 1200 yen and 1800 yen for the second generation, respectively. By contrast, if the DM in the first generation chooses sustainable option B, the sustainable and unsustainable options in the second generation yield 1800 yen and 2400 yen for the second generation, respectively. After the DM made a choice between options A and B, they then proceeded to make the second decision (the intragenerational allocation); the DM divided the payoff from the first decision between themselves and the evaluator. If, for instance, the DM in the first generation chooses option A, the first generation earns 2400 yen, and the DM then divides it between themselves and the evaluator^1^. We would like to note that the DM was introduced as a leader and the evaluator as a member in the scenario.

**TABLE 1:** The payoff in the intergenerational decision in the ISDG.

The 1st group	The 2nd group	The 3rd group	The 4th group	The 5th group	The 6th group	
			A 600 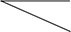	A 0	A −600,	B −1,200
				B −600	A 0,	B −600
		A 1,200 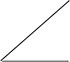	B 0 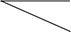	A 600	A 0,	B −600
	A 1,800 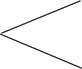			B 0	A 600,	B 0
		B 600 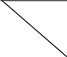	A 1,200 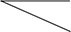	A 600	A 0,	B −600
				B 0	A 600,	B 0
			B 600 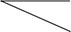	A 1,200	A 600,	B 0
A 2,400 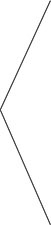				B 600	A 1,200,	B 600
			A 1,200 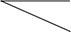	A 600	A 0,	B −600
				B 0	A 600,	B 0
		A 1,800 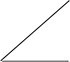	B 600 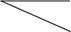	A 1,200	A 600,	B 0
	B 1,200 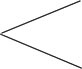			B 600	A 1,200,	B 600
		B 1,200 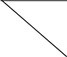	A 1,800 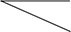	A 1,200	A 600,	B 0
				B 600	A 1,200,	B 600
			B 1,200 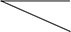	A 1,800	A 1,200,	B 600
				B 1,200	A 1,800,	B 1,200
			A 1,200 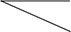	A 600	A 0,	B −600
				B 0	A 600,	B 0
		A 1,800 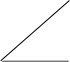	B 600 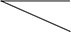	A 1,200	A 600,	B 0
	A 2,400 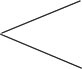			B 600	A 1,200,	B 600
						
		B 1,200 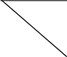	A 1,800 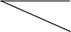	A 1,200	A 600,	B 0
				B 600	A 1,200,	B 600
			B 1,200 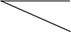	A 1,800	A 1,200,	B 600
B 1,800 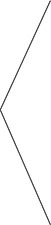				B 1,200	A 1,800,	B 1,200
			A 1,800 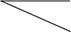	A 1,200	A 600,	B 0
				B 600	A 1,200,	B 600
		A 2,400 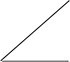	B 1,200 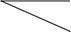	A 1,800	A 1,200,	B 600
	B 1,800 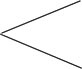			B 1,200	A 1,800,	B 1,200
						
		B 1,800 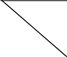	A 2,400 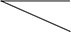	A 1,800	A 1,200,	B 600
				B 1,200	A 1,800,	B 1,200
			B 1,800 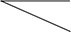	A 2,400	A 1,800,	B 1,200
				B 1,800	A 2,400,	B 1,800

Unit: yen.

**FIGURE 1:**
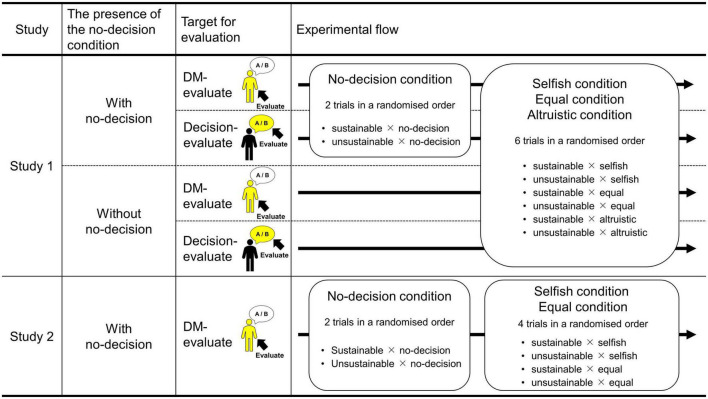
The process of the ISDG evaluation task. The abbreviation “DM” means the decision maker.

In the ISDG evaluation task, participants evaluated the DM as the evaluator. We manipulated the intergenerational decision of the DM (sustainable vs. unsustainable) and the intragenerational allocation of the DM (no-decision vs. altruistic vs. equal vs. selfish), as within-participant factors (see Table 2). Thus, there were eight evaluations to make for participants. In the no-decision condition, participants evaluated the DM who had not yet made an intragenerational decision. Importantly, after participants evaluate the DM in the no-decision condition, they might pay less attention to intragenerational allocations in the subsequent trials. Thus, for half of the participants, we did not present the no-decision conditions (i.e., without no-decision condition). Thus, those in the without no-decision condition made six evaluations, while those in the with no-decision condition made eight (see Figure 1). As we designed the no-decision condition as a baseline, participants in the with no-decision condition were first presented with the no-decision × sustainable and the no-decision × unsustainable conditions in a randomized order and then completed the remaining conditions again in a randomized order. Those in the without no-decision condition completed the six evaluations in a randomized order (see Figure 1). In other words, the presence of the no-decision condition was manipulated as a between-participants factor (with no-decision condition vs. without no-decision condition; Table 3).

**TABLE 2:** The list of the evaluator’s gain in each DM with the intergenerational decision and the intragenerational allocation.

The intergenerational decision	The intragenerational allocation	Money that each player gets as a result of ISDG
Unsustainable	No-decision	Not mentioned (DM had not made intragenerational allocation yet)
	Selfish	DM: ¥1,500, Participant: ¥900
	Equal	DM: ¥1,200, Participant: ¥1,200
	Altruistic (only study 1)	DM: ¥900, Participant: ¥1,500
Sustainable	No-decision	Not mentioned (DM had not made intragenerational allocation yet)
	Selfish	DM: ¥1,200, Participant: ¥600
	Equal	DM: ¥900, Participant: ¥900
	Altruistic (only study 1)	DM: ¥600, Participant: ¥1,200

**TABLE 3:** The summary of the between-participants conditions.

Factor	Each condition name	Explanation
The presence of the no-decision condition	With no-decision	Participants experienced the no-decision condition about the intragenerational allocation. They made 8 evaluations.
	Without no-decision	Participants skipped the no-decision condition about the intragenerational allocation. They made 6 evaluations.
Evaluation target	Decision-evaluate	Participants were asked, “what do you make of the leader’s choice of option A (B)?”
	DM-evaluate	Participants were asked, “what do you think about this leader’s personality?”

In addition, we manipulated the evaluation target as a between-participants factor to explore whether their evaluation differs depending on the domain of the evaluation: the DM or the DM’s decision (Table 3). For participants who were randomly assigned to the decision-evaluate condition, we asked them to indicate how much they thought the decision made by the DM was right, respectable, inappropriate (reversed), harmful (reversed), and how much they supported this decision. Those in the DM-evaluation condition indicated how much they thought the DM was trustworthy, cooperative, generous, likable, kind, reliable, and how much they supported the DM.

### 2.2. Results

We excluded participants who did not seem to pay attention to the study. Namely, we excluded participants who continued to give similar responses to the items measuring personality variables, which we asked for explanatory investigations (see Supplementary material for details about the questionnaires)^2^. This left us 944 participants for analyses. We used the SAS OnDemand for Academics and the HAD 16.0 (Shimizu, 2016) for analyses. All items measuring evaluation were averaged and used as a main dependent variable (decision-evaluate condition: α = 0.85; DM-evaluate condition: α = 0.97). All statistical tests conducted following were two-tailed.

**FIGURE 2:**
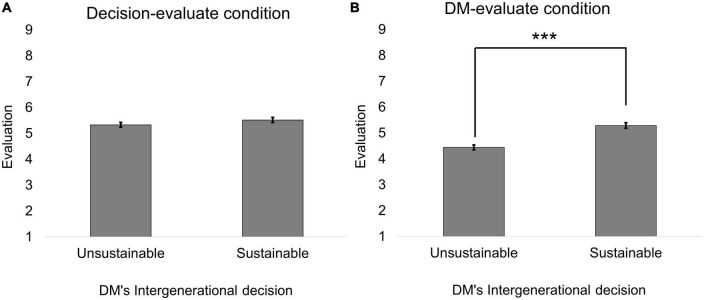
The baseline evaluation in the no-decision condition (Study 1). **(A)** Is the evaluation in the decision-evaluate condition. **(B)** Is the evaluation in the DM-evaluate condition. Error bars represented the standard errors. Different from the main text, sample means were shown. The asterisks indicate Holm-corrected statistical significance based on the simple main effect analysis of the sub-sample. ***p < 0.001.

The average evaluation of each condition is shown in Table 4. First, we conducted a 2 (evaluation target: the DM vs. the DM’s decision) × 2 (intergenerational decision: sustainable vs. unsustainable) ANCOVA^3^ on the evaluation in the no-decision condition (i.e., when there is no information about intragroup allocation) with age and sex as covariates. The reason for separately analyzing this condition was that only half of the total participants, who were assigned to the with no-decision condition, experienced the no-decision condition. Both of the main effect were found to be significant [evaluation target: *F*(1, 466) = 37.268, *p* < 0.001, η*_*G*_^2^* = 0.033; intergenerational decision: *F*(1, 468) = 23.917, *p* < 0.001, η*_*G*_^2^* = 0.028]. The evaluation was more positive when DM chose the sustainable option than when DM chose the unsustainable option (sustainable: *EMM*^4^ = 5.405, *SE* = 0.073; unsustainable: *EMM* = 4.888, *SE* = 0.067). In addition, the evaluation in the decision-evaluate condition was more positive than in the DM-evaluate condition (decision-evaluate: *EMM* = 5.425, *SE* = 0.065; DM-evaluate: *EMM* = 4.868, *SE* = 0.064). The interaction effect was also found to be significant [*F*(1, 468) = 9.727, *p* = 0.002, η*_*G*_^2^* = 0.012]; the simple main effect analysis of the intergenerational decision revealed that the DM who chose the sustainable option was evaluated more positively than the DM who chose the unsustainable option in the DM-evaluate condition [sustainable: *EMM* = 5.291, *SE* = 0.108; unsustainable: *EMM* = 4.445, *SE* = 0.096; *F*(1, 237) = 34.516, Holm-corrected *p* < 0.001, Figure 2A]. However, there was no difference between the sustainable and unsustainable decisions in the decision-evaluate condition *per se* [sustainable: *EMM* = 5.519, *SE* = 0.099; unsustainable: *EMM* = 5.332, *SE* = 0.092; *F*(1, 231) = 1.461, Holm-corrected *p* = 0.228; Figure 2B]. Age was significant, suggesting that older people gave more positive evaluations [*F*(1, 466) = 3.874, *p* = 0.050, η*_*G*_^2^* = 0.004]. There was no significant effect of sex [*F*(1, 466) = 0.661, η*_*G*_^2^* = 0.001, *p* = 0.417].

**TABLE 4:** The descriptive statistics of evaluation in each condition (Study 1).

Intergenerational decision		Unsustainable				Sustainable			
**Intragenerational allocation**		**No-decision**	**Selfish**	**Equal**	**Altruistic**	**No-decision**	**Selfish**	**Equal**	**Altruistic**
With no-decision	DM-evaluate (*N* = 238)	4.44 (1.48)	4.30 (1.52)	5.23 (1.56)	5.32 (1.51)	5.29 (1.65)	4.25 (1.59)	5.39 (1.64)	5.46 (1.62)
	Decision-evaluate (*N* = 232)	5.33 (1.41)	4.91 (1.30)	5.57 (1.41)	5.46 (1.39)	5.52 (1.50)	4.90 (1.50)	5.58 (1.46)	5.48 (1.27)
Without no-decision	DM-evaluate (*N* = 238)	−	4.17 (1.56)	5.33 (1.66)	5.34 (1.60)	−	4.38 (1.66)	5.44 (1.71)	5.58 (1.66)
	Decision-evaluate (*N* = 236)	−	4.72 (1.34)	5.45 (1.27)	5.38 (1.19)	−	4.73 (1.35)	5.53 (1.33)	5.45 (1.20)

Values in parentheses are standard deviations.

**FIGURE 3:**
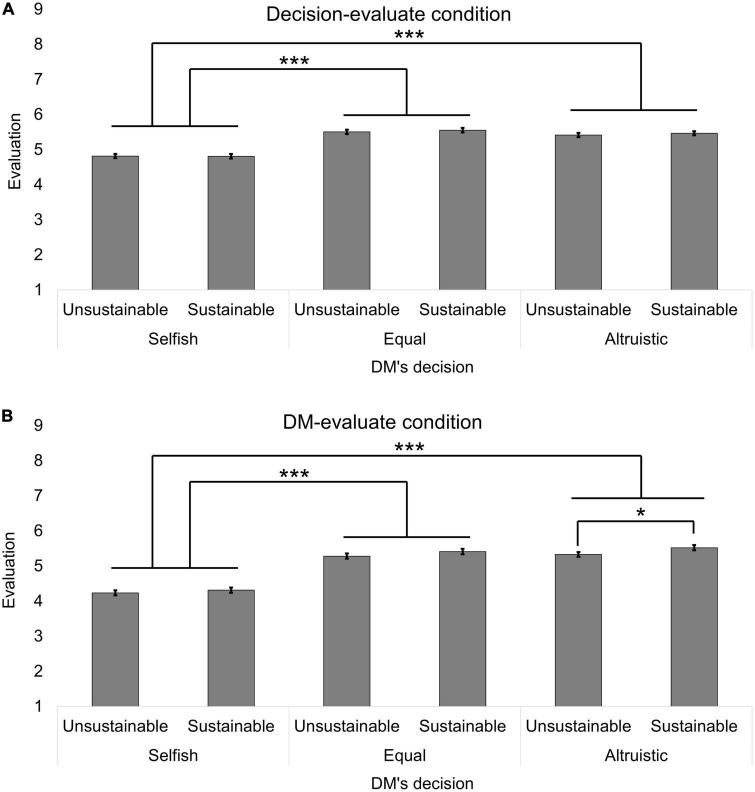
The evaluation in the three intragenerational allocation conditions other than the no-decision condition (Study 1). **(A)** Is the evaluation in the decision-evaluate condition. **(B)** Is the evaluation in the DM-evaluate condition. Error bars represented the standard errors. Different from the main text, sample means were shown. The asterisks indicate Holm-corrected statistical significance based on the simple main effect analysis of the sub-sample. *p < 0.05, ***p < 0.001.

Next, we conducted a 2 (evaluation target: the DM vs. the DM’s decision) × 2 (intergenerational decision: sustainable vs. unsustainable) × 3 (intragenerational allocation: selfish vs. equal vs. altruistic) ANCOVA with age, sex, and the presence of the no-decision condition (with no-decision vs. without no-decision) as covariates^5^ (Figure 3). All the main effects were significant [evaluation target: *F*(1, 939) = 16.943, *p* < 0.001, η*_*G*_^2^* = 0.007; intergenerational decision: *F*(1, 942) = 4.583, *p* = 0.033, η*_*G*_^2^* = 0.001; and intragenerational allocation: *F*(2, 1884) = 217.118, *p* < 0.001, η*_*G*_^2^* = 0.075]. Again, the evaluation was more positive when DM chose the sustainable option than when DM chose the unsustainable option (sustainable: *EMM* = 5.181, *SE* = 0.035; unsustainable: *EMM* = 5.099, *SE* = 0.036). The evaluation in the decision-evaluate condition were more positive than in DM-evaluate condition (decision-evaluate: *EMM* = 5.263, *SE* = 0.042; DM-evaluate: *EMM* = 5.018, *SE* = 0.042). In addition, the selfish allocation among the current generation was evaluated as being significantly more negative than other allocations according to Holm-method multiple comparisons (selfish: *EMM* = 4.546, *SE* = 0.042; equal: *EMM* = 5.440, *SE* = 0.040; altruistic: *EMM* = 5.434, *SE* = 0.042). The evaluation target × intragenerational allocation interaction was significant [*F*(2, 1884) = 14.610, *p* < 0.001, η*_*G*_^2^* = 0.005]. Yet, the other interaction terms were not significant (*F_*s*_* < 1.796, *p*_*s*_ > 0.181, η*_*G*_^2^_*s*_* < 0.001). We probed the significant interaction and found that the evaluation in the DM-evaluate condition was more negative than in the decision-evaluate condition when the intragenerational allocation was selfish [decision-evaluate: *EMM* = 4.815, *SE* = 0.059; DM-evaluate: *EMM* = 4.276, *SE* = 0.059; *F*(1, 939) = 41.478, Holm-corrected *p* < 0.01] and equal [decision-evaluate: *EMM* = 5.531, *SE* = 0.057; DM-evaluate: *EMM* = 5.349, *SE* = 0.057; *F*(1, 939) = 5.107, Holm-corrected *p* = 0.048]. However, this difference was not significant when the intragenerational allocation was altruistic [decision-evaluate: *EMM* = 5.442, *SE* = 0.059; DM-evaluate: *EMM* = 5.427, *SE* = 0.059; *F*(1, 939) = 0.031, Holm-corrected *p* = 0.860]. No covariates had significant effects in this analysis [age: *F*(1, 939) = 2.291, η*_*G*_^2^* = 0.001, *p* = 0.130; sex: *F*(1, 939) = 0.598, η*_*G*_^2^* = 0.000, *p* = 0.439; the presence of the no-decision condition: *F*(1, 939) = 0.225, η*_*G*_^2^* = 0.000, *p* = 0.635].

In summary, future altruism was more positively evaluated compared to unsustainable behaviors, even if future altruism decreased the current generation’s benefit. In addition, selfish intragenerational allocation was evaluated more negatively than equal and altruistic allocation. However, the interaction between intergenerational decision and intragenerational allocation was not significant. Interestingly, the effect size of the intergenerational decision was larger in the ANCOVA of no-decision condition than in the ANCOVA of the other three intragenerational allocation conditions. This suggests that the display of intragenerational allocation decreased the evaluation difference between the evaluations of sustainable and unsustainable behaviors. Comparing the effect size of the main effects between intergenerational decision and intragenerational allocation, intragenerational allocation affected the evaluation more strongly than the intergenerational decision when the intragenerational allocation was revealed. Regarding the evaluation target, the evaluation in the DM-evaluate condition was more negative than in the decision-evaluate condition. Especially, the evaluation of the DM became more negative than that of the decision when the DM made self-serving decisions (unsustainable decisions in the intergenerational decision and selfish allocations in the intragenerational allocation). As a result, the difference in the evaluation between the sustainable and unsustainable decisions was larger in the DM-evaluate condition.

Why was future altruism positively evaluated, even if it may be costly to current others, including the evaluators themselves? We focused on the result that the main effect of intergenerational decision was larger in the no-decision condition, where the DM’s altruism toward current others was not revealed. This led us to further predict that people may evaluate future altruists based on their altruistic tendencies for the current generation, but when their altruistic tendencies are unknown, they generally positively evaluate future altruists, assuming that future altruists would not behave selfishly toward the current generation. When people do not know about how others would treat them and their generation, people may use future altruism as a proxy to infer their altruistic tendencies. If so, a positive evaluation of future altruism should be mediated by high estimations of altruism toward current others. To test the hypothesis, we conducted Study 2. To this end, in Study 2, we focused on the evaluation in the DM-evaluate condition, which had a larger difference between the sustainable and unsustainable decisions, and investigated whether the estimations of altruism toward current others explained this difference.

## 3. Study 2

### 3.1. Method

#### 3.1.1. Participants

The survey form was prepared in Japanese and participants were recruited through the same research company as study 1. We requested this company to ensure that people who had participated in Study 1 did not participate again.

In Study 2, we sought to recruit at least 250 participants, which is the number of participants that we had in each between-subject condition in Study 1. Therefore, the target sample size was initially set at 275 so that 250 participants would remain after the exclusion. This sample size was calculated based on the exclusion rate in Study 1 (5.6%). However, based on advice from the research company, the sample size was later set to 300, a convenient number for generating an equal allocation of gender and age. Therefore, we collected 300 participants (150 men and 150 women; *M*_*age*_ = 45.08, *SD* = 15.06).

We conducted preregistration for Study 2 using the Open Science Framework (see Acknowledgments^6^). We conducted this study according to the preregistration, but the sample size was changed, as mentioned above. There are no other changes in the preregistration.

The ethics committee of Kochi University of Technology approved this study’s procedure, which met the requirements of the Declaration of Helsinki. We obtained informed consent from all participants; the description of this survey was displayed on the first page, and participants started the survey only after they agreed to participate.

#### 3.1.2. Procedure

The procedure of Study 2 was similar to that of Study 1. However, there were four main changes to the ISDG evaluation task to measure expectation about how altruistic future altruists are to those in the current generation. First, we measured the estimation of the DM’s allocation among the current generation in the no-decision condition. In particular, we asked participants how much money they thought the DM allocated to them. Participants answered this question in increments of 100 yen. Second, we removed the manipulation of the presence of the no-decision condition, and all participants experienced the no-decision condition to estimate DM’s allocation. In Study 1, we found that this did not affect the evaluation of future altruism. Third, we also removed the manipulation of the evaluation target, and all participants evaluated the DM’s impression in this study. This was because it seemed to be convenient to investigate the mediation effect to focus on the condition that has the larger difference in the evaluation between sustainable and unsustainable decisions. Finally, we removed the altruistic condition from the DM’s intragenerational allocation because the evaluation was similar to that in the equal intragenerational allocation in Study 1.

In addition, we added two demographic questions to the end of the survey as control variables: parenthood and grandparenthood. This was because we suspected that having children or grandchildren may increase the concern for the future generation and also affect the evaluation of future altruism.

### 3.2. Results

We excluded participants who did not seem to pay attention to the study. Namely, we excluded participants who continued to give similar responses to the items measuring personality variables, which we asked for exploratory investigations (see Supplementary material for details about the questionnaires)^7^. This left us 293 participants for analyses.

**FIGURE 4:**
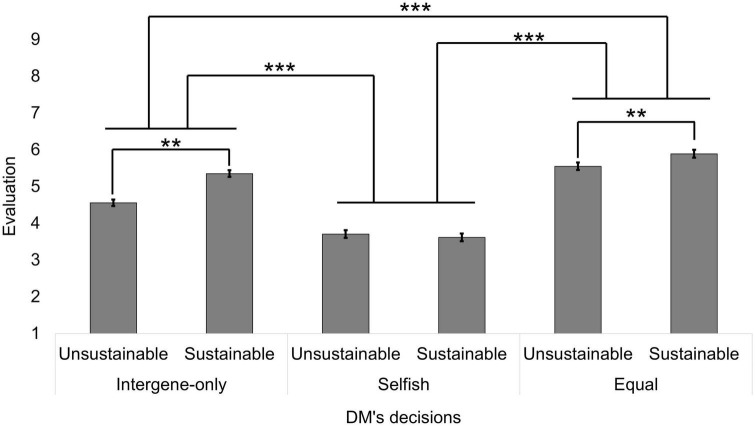
The evaluations in Study 2. The error bars represented the standard errors. Different from the main text, sample means were shown. The asterisks indicate statistical significance based on the ANCOVA and the simple main effect analysis of the sub-sample (Holm-corrected). **p < 0.01, ***p < 0.001.

We summarize descriptive statistics of the average evaluation score for each condition in Figure 4. First, we conducted a 2 (intergenerational decision: sustainable vs. unsustainable) × 3 (intragenerational allocation: no-decision vs. selfish vs. equal) ANCOVA^8^. The covariates were age, gender, parenthood, and grandparenthood. As a result, both of the main effects were significant [intergenerational decision: *F*(1, 292) = 27.084, *p* < 0.001, η*_*G*_^2^* = 0.010; intragenerational allocation: *F*(2, 584) = 183.446, *p* < 0.001, η*_*G*_^2^* = 0.198]. As in Study 1, the evaluation of the sustainable DM was more positive than of the unsustainable DM (sustainable: *EMM* = 4.953, *SE* = 0.068; unsustainable: *EMM* = 4.603, *SE* = 0.073). In addition, there were significant differences between all intragenerational allocation according to multiple comparisons (Holm method); the evaluation in the equal allocation condition was the highest; the second highest was the no-decision condition; and it was the lowest in the selfish allocation condition (no-decision: *EMM* = 4.954, SE = 0.070; selfish: *EMM* = 3.660, *SE* = 0.098; equal: *EMM* = 5.720, *SE* = 0.096). The interaction effect was also significant [*F*(2, 584) = 29.994, *p* < 0.001, η*_*G*_^2^* = 0.011]. The simple main effect analysis of intergenerational decision revealed that the DM who chose the sustainable option was evaluated more positively than who chose the unsustainable option in the no-decision condition [sustainable: *EMM* = 5.352, *SE* = 0.092; unsustainable: *EMM* = 4.556, *SE* = 0.088; *F*(1, 292) = 48.436, Holm-corrected *p* < 0.01] and equal allocation condition [sustainable: *EMM* = 5.890, *SE* = 0.106; unsustainable: *EMM* = 5.550, *SE* = 0.101; *F*(1, 292) = 16.032, Holm-corrected *p* < 0.01]. In the selfish condition, the effect of the intergenerational decision was insignificant [sustainable: *EMM* = 3.616, *SE* = 0.105; unsustainable: *EMM* = 3.704, *SE* = 0.103; *F*(1, 292) = 1.249, Holm-corrected *p* = 0.265]. The effect of covariates were all insignificant [age: *F*(1, 288) = 0.008, η*_*G*_^2^* = 0.000, *p* = 0.929; sex: *F*(1, 288) = 0.010, η*_*G*_^2^* = 0.000, *p* = 0.919; parenthood: *F*(1, 288) = 0.003, η*_*G*_^2^* = 0.000, *p* = 0.958; grandparenthood: *F*(1, 288) = 0.461, η*_*G*_^2^* = 0.001, *p* = 0.498].

**FIGURE 5:**
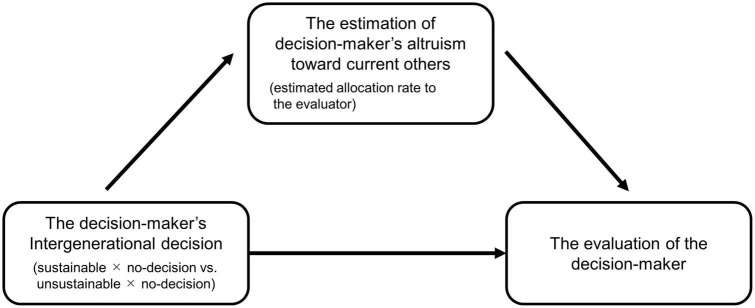
The mediation model investigated in Study 2.

Next, we investigated the mediation effect of the estimation of how altruistic a DM is toward those in the current generation (see Figure 5). We conducted a two-condition within-participants statistical mediation analysis (Montoya and Hayes, 2017). We did not add any covariates because covariates were to be considered only when they were predicted to affect differently in each condition in this analysis (Montoya, 2019), and we did not expect this difference. We used a SAS macro, MEMORE (Montoya, 2019), to estimate the total, direct, and indirect effects in the model. This macro estimates the confidence interval for these effects using the bootstrap method. We calculated the 95% confidence intervals with 10,000 bootstrap samples in our analysis. If they did not include 0, we considered the effect significant.

**FIGURE 6:**
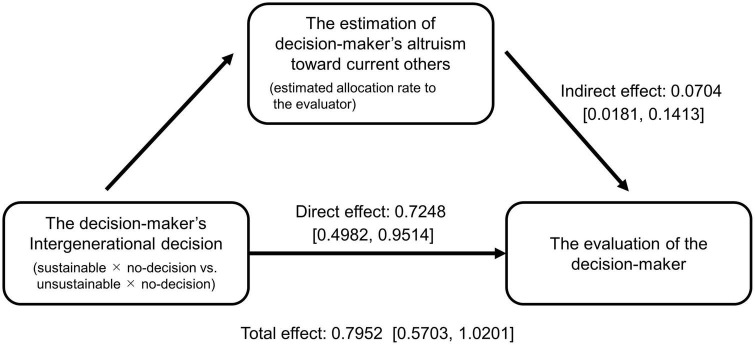
The result of the mediation analysis in Study 2. The values in the brackets are the confidence intervals.

The result revealed that both the direct and indirect effects were significant; their confidence intervals did not contain zero (Figure 6). However, the indirect effect was much smaller than the direct effect. As such, the effect of the estimation of the DM’s altruism toward current others was insufficient to explain why future altruism is positively evaluated, although the DM who chose the sustainable option was predicted to be more altruistic toward current others than the DM who chose the unsustainable option (estimated allocation rate to evaluator: sustainable: 48.1%; unsustainable: 45.5%; Wilcoxon signed-rank test: *S* = 3227, *p* < 0.001).

## 4. Discussion

Our studies suggest that people positively evaluate future altruism, even if future altruism reduces the payoff for their own generation as well as themselves. We also found that the evaluation difference between the sustainable and unsustainable DM was the largest when the DM’s altruism toward current others was not revealed. By contrast, the effect of the intergenerational decision on the evaluation greatly diminished when the DM’s intragenerational allocation was revealed. In this case, intragenerational allocation, namely altruism toward current others, had a stronger influence on the evaluation; the DM who allocated resources selfishly among the current generation was more negatively evaluated than the DM who allocated resources equally or altruistically.

Regardless, Study 2 suggests that the positive evaluation of future altruism does not stem from the prediction that future altruists will also de facto be altruistic toward current others. The prediction of altruism toward current others had only a small indirect effect on the DM’s evaluation. This means that the good evaluation of future altruists comes from future altruism itself. From this perspective, people may distinguish the altruism displayed toward current others from that displayed toward future others for evaluation. However, our studies could not identify exactly why people positively evaluated future altruism. Further research is needed on this point.

We would like to note that the ecological validity of this study is limited. In the ISDG game, we defined other players who will play the game in later rounds as “future generations.” This game represents important features of future altruism such as the unidirectionality of resource flow and the conflict of interest between the current and future generations. Yet, while everyday future altruism is often directed toward those who are not yet born (i.e., future generation), recipients of future altruism in our studies are other participants. In other words, our design did not fully reflect one feature of future altruism, that is, altruism being toward those not yet born. Therefore, future studies should examine the evaluation of future altruism in more ecologically suitable contexts, for instance, by focusing on pro-environmental behaviors (e.g., supporting the introduction of carbon tax).

There were some other limitations to these studies. First, our studies focused on second-party evaluations of future altruism, but the evaluation from the third party, who does not incur the cost of future altruism, has not yet been examined. Because we assumed that evaluators incurring costs of future altruists’ behavior would lead to negative evaluation, we did not focus on the evaluation from the third party in our studies. Our results suggest that people positively evaluate future altruism even when they have to incur its cost. Therefore, it is expected that the third party with no interest will also positively evaluate future altruism, but it needs to be confirmed. Second, the participants may have been already altruistically biased according to the measure of personality, social value orientation (see Supplementary Tables S7, S8 and Supplementary Figure S1). It needs to be checked whether our results can be replicated with a potentially less biased sample. Third, we conducted two studies in vignettes because we needed to manipulate the target decisions. Therefore, our results should be validated in situations where participants are fully incentivized. Finally, our result that future altruism was evaluated positively raises a question about the hypothesis that the costs of the current others discourage future altruism. We predicted that people behave altruistically toward future generations in IGG (Hauser et al., 2014; Lohse and Waichman, 2020; Klaser et al., 2021) more than in ISDG (Kamijo et al., 2017; Shahrier et al., 2017) because future altruism is not supported by current others when the given behavior is costly for current others. However, our result suggested that current others support future altruism even if they have to incur costs. In this case, why are the degrees of altruistic behavior toward future generations different between these games? Is future altruism disadvantageous to the actual interests of the future altruist, even if a good reputation is obtained? Or are there other factors? Further research is needed on these questions.

In summary, we investigated whether future altruism leads positive or negative reputation to reveal whether there is a reputational disadvantage for future altruism. Our studies showed that future altruist is positively evaluated by current others. This suggests the possibility that future altruism is promoted by rewards from current others, as well as that altruism toward current third parties (indirect reciprocity). It is a plausible assumption that positive and negative evaluations induce rewards and punishments, respectively. Although it is necessary to examine whether future altruism can be maintained by punishments or rewards from current others, it is an important suggestion that reputational incentives may promote future altruism. If so, self-serving individuals may behave altruistically when the reputational incentives are emphasized. There may be both preference-based and reputation-based mechanisms behind future altruism (Inoue et al., 2021). The reputation-based mechanisms may promote future altruism especially among those who do not have a prosocial preference toward future generations. Highlighting reputational benefits of future altruism, i.e., disclosing the amount of contribution, may be an effective way to encourage sustainable behaviors.

## Data availability statement

The datasets presented in this study can be found in online repositories. The names of the repository/repositories and accession number(s) can be found below: https://osf.io/qkfhm.

## Ethics statement

The studies involving human participants were reviewed and approved by the Ethics Committee of Kochi University of Technology. Written informed consent for participation was not required for this study in accordance with the national legislation and the institutional requirements.

## Author contributions

YI and NM developed the research concept, created the questionnaire, conducted the data analysis, and drafted the manuscript. TS provided critical revisions. All authors approved the final version of the manuscript.

## Footnotes

1 We did not inform participants about whether the DM knew if they would be evaluated.

2 We used a questionnaire for social value orientation (Eek and Gärling, 2006), which measures resource allocation preference. In this questionnaire, a person with a somewhat consistent preference will choose various options throughout the whole questionnaire because an option corresponding to a particular preference is different for each question. Therefore, we excluded those who chose the same options for all questions in this questionnaire, regarding this as paying insufficient attention to the survey. Note that the main results were similar when we analyzed all participants (there was no difference in results except that the effect of age was no longer significant in the ANCOVA of no-decision condition).

3 As many studies assume a normal distribution about the evaluation measured by questionnaires, we also assumed normal distributions. However, actual distributions of some indexes have higher kurtoses than the normal distribution. We reported the result of the ANCOVA test because the main results were almost the same as the analyses assuming other distributions (see Supplementary material).

4 Estimated mean when covariates equal mean values are shown.

5 We conducted an analysis where we included the presence of the no-decision condition as an independent variable and examined the interaction effect between this and other independent variables. However, there was no interaction effect (see Supplementary Table S1). Therefore, we used a simpler model where this factor was a covariate.

6
https://osf.io/cwfxj

7 We again used a questionnaire for social value orientation (slider measure; Murphy et al., 2011). Although the questionnaire differed from Study 1, it is the same in that an option corresponding to a particular preference is different for each question. We excluded those who chose the same options for all questions in this questionnaire. Note that all the results were similar when we analyzed all participants.

8 As many studies assume a normal distribution about the evaluation measured by questionnaires, we also assumed a normal distribution on the occasion of the preregistration. However, when the actual distribution was checked ex-post, kurtoses of some measures were higher than the normal distribution. Although the reliability of the ANCOVA test was decreased, we reported the result of the ANCOVA test as we preregistered. This is because other major continuous probability distributions also did not apply to the actual distributions. The main results were the same as the analyses assuming other distributions (see Supplementary material).
