# Supplementary material for: Positive reputation for altruism toward future generations regardless of the cost for current others
Source: Front Psychol. 2023 Jan 24;13:895619. doi: 10.3389/fpsyg.2022.895619 (PMC9902652; doi:10.3389/fpsyg.2022.895619)
Supplement: Supplementary file 1 [file Data_Sheet_1.pdf]

Supplementary Material

Supplementary Table in the main text

Supplementary Table S1. The ANCOVA of the other than no-decision condition in Study 1 when the presence of the no-decision condition was an independent variable

Variables	SS	MS	MSe	η_G^2	*F*	df1	df2	*p*
Intergenerational decision (InterD)	9.559	9.559	2.093	.001	4.568	1	940	.033*
Intragenerational allocation (IntraA)	1000.230	500.115	2.308	.075	216.685	2	1880	.000**
Evaluation target (Target)	85.247	85.247	5.013	.007	17.004	1	938	.000**
The presence of the no-decision condition (Presence)	1.184	1.184	5.013	.000	0.236	1	938	.627
InterD × IntraA	1.750	0.875	0.716	.000	1.222	2	1880	.294
InterD × Target	3.768	3.768	2.093	.000	1.800	1	940	.180
InterD × Presence	1.794	1.794	2.093	.000	0.857	1	940	.355
IntraA × Target	67.485	33.742	2.308	.005	14.620	2	1880	.000**

Variables	SS	MS	MSe	η_G^2	*F*	df1	df2	*p*
IntraA × Presence	2.893	1.446	2.308	.000	0.627	2	1880	.499
Target × Presence	8.806	8.806	5.013	.001	1.756	1	938	.185
InterD × IntraA × Target	0.267	0.134	0.716	.000	0.187	2	1880	.822
InterD × Target × Presence	0.274	0.274	2.093	.000	0.131	1	940	.717
InterD × IntraA × Presence	0.937	0.469	0.716	.000	0.655	2	1880	.514
IntraA × Target × Presence	0.161	0.081	2.308	.000	0.035	2	1880	.939
InterD × IntraA × Target × Presence	1.683	0.841	0.716	.000	1.176	2	1880	.308
Age	11.512	11.512	5.013	.001	2.296	1	938	.130
Sex	3.066	3.066	5.013	.000	0.612	1	938	.434

Supplementary Materials 1:

Reanalysis of the result of the main text using non-normal distribution

In psychology, the distribution of the answer to the questionnaire is often assumed to be the normal distribution. Therefore, we mainly used ANCOVA in the analysis. However, a posteriori, we found that the evaluations measured by the questionnaires were not normally distributed. We reported the result of ANCOVA in the main text according to the preregistration because any other major continuous probability distribution (gamma, log-normal) did not fit the actual distribution. Here we reported the result of another statistical method, the generalized linear mixed model (GLMM) for validity. We adopted the log-normal distribution in all GLMMs because this model had the least AIC among three continuous probability distributions (normal distribution, gamma distribution, and log-normal distribution). The link function was the identity function. We used the glimmix procedure of SAS OnDemand for Academics, the statistical software, in these analyses. The parameters were estimated using the Gauss-Hermite quadrature.

1.1 Study 1

First, we reanalyzed the evaluation in the no-decision condition by GLMM. In this analysis, intergenerational decision, evaluation target, sex, age (centered in prior), and the interaction between intergenerational decision and the evaluation target was put as the fixed effects. The random effect was the participants' serial numbers. We have to note that the covariance parameter estimate of the random effect was zero in this model. Although the random effects were left in the model based on the survey design, the model estimations were the same as those without the random effects (cf. Kiernan et al., 2012).

The result was displayed in Supplementary Table S2. Results were almost the same as the results of the ANCOVA reported in the main text. First, the main effect of the intergenerational decision and the evaluation target was significant. The evaluation was more positive when DM chose the sustainable option than when DM chose the unsustainable option. In addition, the evaluation was more positive in the decision-evaluate condition than in the DM-evaluate condition. Second, the interaction between the intergenerational decision and the evaluation target was also significant; in the decision-evaluate condition, the difference in the evaluation between sustainable and unsustainable decisions was small.

However, there was one difference between the result of GLMM and ANCOVA; the age of the participants did not have a significant effect in the GLMM, although it had a significant effect in the ANCOVA.

Supplementary Table S2.

The result of GLMM in the no-decision condition of Study 1.

Effect	Estimates	SE	CI	df	T	p
**Intercept**	1.4092	0.02708	[1.356,1.462]	466	52.03	<.0001
**Intergenerational decision (InterD)**						
**Sustainable**	0.1964	0.03439	[0.129, 0.264]	468	5.71	<.0001
**Unsustainable**	0		Contrast			
**Evaluation target (Target)**						
**Decision-evaluate**	0.2217	0.03461	[0.154, 0.290]	466	6.41	<.0001
**DM-evaluate**	0		Contrast			
**InterD × Target**						
**Sustainable×Decision**	-0.1639	0.04894	[-0.260, -0.068]	468	-3.35	0.0009
**Sex**						
**Female**	0		Contrast			
**Male**	0.001594	0.02448	[-0.047, 0.050]	466	0.07	0.9481
**Age**	0.000994	0.000825	[-0.001, 0.003]	466	1.20	0.2289
**AIC (Akaike's information criteria)**	1380.80					

Next, we reanalyzed the evaluation in the other three intragenerational allocation conditions. In this analysis, intergenerational decision, intragenerational allocation, the evaluation target, and these interactions in addition to sex, age (centered in prior), and the presence of the no-decision condition were put as the fixed effects. The random effect was the participants' serial numbers.

The result was displayed in Supplementary Table S3. Unlike the ANCOVA test, the main effect of the intergenerational decision was not significant, whichever intrageneration allocation was set as the contrast. This result was consistent with the weak effect of intergenerational decisions on the evaluation.

Other effects were the same with the ANCOVA test. The main effects of intragenerational allocation and the evaluation target were significant. The selfish intragenerational allocation was negatively evaluated than equal and altruistic allocations, and the evaluation was more positive in the decision-evaluate condition than in the DM-evaluate condition. In addition, the interaction between intragenerational allocation and the evaluation target was significant; the evaluation in the DM-evaluate condition was more negative than in the decision-evaluate condition when the intragenerational allocation was selfish, but not when the intragenerational allocation was equal or altruistic. The other interaction terms were not significant.

Supplementary Table S3.

The result of GLMM of the three intragenerational allocation condition (selfish, equal, altruistic) of Study 1

Effect	Estimates	SE	CI	df	T	p
**Intercept**	1.3556	0.02055	[1.315, 1.396]	939	65.97	<.0001
**Intergenerational decision (InterD)**						
**Sustainable**	0.01572	0.02065	[-0.025, 0.056]	942	0.76	0.4466
**Unsustainable**	0		Contrast			
**Intragenerational allocation (IntraA)**						
**Selfish**	0		Contrast			
**Equal**	0.2565	0.02065	[0.216, 0.297]	1884	12.42	<.0001
**Altruistic**	0.2731	0.02065	[0.233, 0.314]	1884	13.23	<.0001
**Evaluation target (Target)**						
**Decision-evaluate**	0.1694	0.02459	[0.121, 0.218]	939	6.89	<.0001
**DM-evaluate**	0		Contrast			
**InterD × IntraA**						
**Sustainable×Equal**	0.007929	0.02920	[-0.049, 0.065]	1884	0.27	0.7860
**Sustainable×Altruistic**	0.01527	0.02920	[-0.042, 0.073]	1884	0.52	0.6011
**InterD × Target**						

Effect	Estimates	SE	CI	df	T	p
**Sustainable×Decision**	-0.02639	0.02933	[-0.084, 0.031]	942	-0.90	0.3685
**IntraA × Target**						
**Equal×Decision**	-0.09924	0.02933	[-0.157, -0.042]	1884	-3.38	0.0007
**Altruistic×Decision**	-0.1306	0.02933	[-0.188, -0.073]	1884	-4.45	<.0001
**InterD × IntraA × Target**						
**Sustainable×Equal×Decision**	0.005534	0.04148	[-0.076, 0.087]	1884	0.13	0.8939
**Sustainable×Altruistic×Decision**	0.008729	0.04148	[-0.073, 0.090]	1884	0.21	0.8333
**Sex**						
**Female**	0		Contrast			
**Male**	-0.02380	0.01570	[-0.055, 0.007]	939	-1.52	0.1299
**Age**	0.000278	0.000527	[-0.001, 0.001]	939	0.53	0.5976
**Presence of the no-decision condition**						
**With no-decision**	0		Contrast			
**Without no-decision**	0.005063	0.01570	[-0.026, 0.036]	939	0.32	0.7471
**AIC (Akaike's information criteria)**	4315.34					

1.2 Study 2

We conducted GLMM to recheck the effect of the intergenerational decision and intragenerational allocation on the evaluation. In this analysis, intergenerational decision, intragenerational allocation, and the interaction between them were put as the fixed effects in addition to sex, age (centered in prior), parenthood, and grandparenthood. The random effect was the participants’ serial numbers.

The result displayed in Supplementary Table S4 was the same as the ANCOVA test. The main effect of the intergenerational decision and the intragenerational allocation were significant. The evaluation of the sustainable DM was more positive than the unsustainable DM when the no-decision condition was set to contrast. The evaluation in the selfish intragenerational allocation condition was more negative than in the no-decision condition, and the evaluation in the equal intragenerational condition was more positive in the no-decision condition. In addition, the interaction between the intergenerational decision and intragenerational allocation was also significant; when the intragenerational allocation was selfish or equal, the evaluation difference between sustainable and unsustainable intergenerational decisions was very small.

Supplementary Table S4. The result of GLMM in Study 2

Effect	Estimates	SE	CI	df	T	p
**Intercept**	1.4409	0.04041	[1.361, 1.520]	288	35.66	<.0001
**Intergenerational decision (InterD)**						
**Sustainable**	0.1771	0.03312	[0.112, 0.242]	292	5.35	<.0001
**Unsustainable**	0		Contrast			
**Intragenerational allocation (IntraA)**						
**No-decision**	0		Contrast			
**Selfish**	-0.2828	0.03312	[-0.348, -0.218]	584	-8.54	<.0001
**Equal**	0.2070	0.03312	[0.142, 0.272]	584	6.25	<.0001
**InterD × IntraA**						
**Sustainable×Selfish**	-0.2096	0.04683	[-0.302, -0.118]	584	-4.47	<.0001
**Sustainable×Equal**	-0.1160	0.04683	[-0.208, -0.024]	584	-2.48	0.0135
**Sex**						
**Female**	0		Contrast			
**Male**	0.01483	0.03747	[-0.059, 0.089]	288	0.40	0.6925
**Age**	0.001401	0.001531	[-0.002, 0.004]	288	0.92	0.3607

Effect		Estimates	SE	CI	df	T	p
**Parenthood**							
	**Yes**	-0.00414	0.04225	[-0.087, 0.079]	288	-0.10	0.9219
	**No**	0		Contrast			
**Grandparenthood**							
	**Yes**	-0.04738	0.06754	[-0.180, 0.086]	288	-0.70	0.4836
	**No**	0		Contrast			
**AIC (Akaike's information criteria)**		2190.34					

Supplementary Materials 2:

The instructions for the Intergenerational Sustainability Dilemma Game (ISDG) evaluation task (original language: Japanese)

The money allocation task

(1) The overview of the task

There are 12 participants in this experiment, including you, at the same time.

First, six pairs, from the first pair to the sixth pair, will be established by lottery in this task. One person in each pair will be the "leader," and the other will be the "member." These roles are randomly assigned.

The leader will make two decisions. How much money the participants earn will be determined by these decisions.

The first decision will determine the total amount of money earned by the pair.

The second decision will determine how the money will be divided between the pair. This decision eventually fixes the amount of money accruing to the leader and the member of each pair.

(2) The first decision: determination of the total amount of money earned by the pair

The first decision is made in order, one pair at a time, from the first pair to the sixth pair. Each pair's decision affects the amount of money that accrues to the subsequent pairs.

First, the leader of the first pair chooses between option A and option B. The amount of money earned will be determined as shown in the linked table[1] depending on this choice. Please see the table.

As the table shows, if the leader chooses option A, the first pair will get 2400 yen.
If the leader chooses option B, the first pair will get 1800 yen.

However, the decision of the first pair affects the amount of money subsequent pairs will earn.

If the first pair chooses option A, the amount of money for both options decreases by 600 yen at the time of the second pair's decision. In other words, the leader of the second pair chooses whether they earn 1800 yen (option A) or 1200 yen (option B) if the first pair have chosen option A.

However, if the first pair chooses option B, the amount of money for both options will not change at the time of the second pair's decision. In other words, the leader of the second pair chooses whether they earn 2400 yen (option A) or 1800 yen (option B) again if the first pair chooses option B.

---

[1] Table 1 in the main text was displayed in the link.

First pair	Second pair
A　¥2400	A　¥1800
	B　¥1200
B　¥1800	A　¥2400
	B　¥1800

Next, let us think about the second pair’s decision when the first pair chose option A.

If the second pair chooses option A, the amount of money for both options for the third pair decreases by a further 600 yen, and the third pair will have to choose between getting themselves 1200 yen (option A) or 600 yen (option B).

On the other hand, if the second pair chooses option B, the third pair will get 1800 yen if they choose option A and 1200 yen if they choose option B.

First pair	Second pair	Third pair
A ¥2400	A ¥1800	A ¥1200
		B ¥600
	B ¥1200	A ¥1800
		B ¥1200
B ¥1800	A ¥2400	A ¥1800
		B ¥1200
	B ¥1800	A ¥2400
		B ¥1800

In summary, the amount of money obtained in the subsequent pairs will decrease by 600 yen if a leader of a pair routinely chooses option A.

On the other hand, the amount of money obtained by the subsequent pairs will be the same as the current pair if the leader of a pair chooses option B.

Please look at the linked table carefully.

At this decision, the amount of money the pair can get will be communicated to the pair.

The preceding pair's decisions determine these amounts.

(However, in the case of the first pair, the amount is fixed at 2400 yen if they choose A and 1800 yen if they choose B.)

Depending on the previous pair’s choice, the amount of money accruing to each option may be less than zero.

(3) The second decision: the allocation of money between the pair

After the leader chooses option A or option B, they decide how to allocate the earned money within that pair. If the earned money is actually less than zero, participants in that pair have to pay for it, so the leader decides how to allocate that burden between them.

At this decision, only the leader’s decision will determine the final amount of money accruing to that pair. The member will receive the money as decided by the leader.

Supplementary Materials 3:

The confirmation questions and correct answers (original language: Japanese)

The confirmation questions

Please choose the correct answer for each question.

A brief explanation about the task and a table of the amount of money earned can be found here[2].

If you answer many questions incorrectly, you may not be able to participate in this survey, so please check the table carefully before answering.

Q1. Which decision will bring a larger amount of money to the first pair, option A or B?

1. option A
2. option B

(Correct answer: option A)

Q2. If the leader of the first pair chooses option A, how will be the amount of money the second pair earn by the decision compared to the first pair?

1. increases

[2] Table 1 in the main text and the summary of the explanation about the ISDG evaluation task were displayed in the link.

2. does not change
3. decreases

(Correct answer: decreases)

Q3. If the leader of the first pair chooses option B, how much will be the amount of money the second pair earns by the decision compared to the first pair?

1. increases
2. does not change
3. decreases

(Correct answer: does not change)

Supplementary Materials 4:

The statistical analysis containing post questionnaires used in two studies

To explanatorily examine the effect of personality on the evaluation of altruism toward future generations (future altruism), we measured participants' personalities. We used personality scales in Supplementary Table S5. We also asked participants how they would make the intergenerational decision and intragenerational allocation if they had been a DM.

The mean and standard deviation of the score of each questionnaire is represented in Supplementary Table S6. The percentage of each Social Value Orientation (SVO) is represented in Supplementary Tables S7 and S8. The distribution of Social Value Orientation in Study 2, which measured by Slider Measure (Murphy et al., 2011), is represented in Figure S1. Finally, the percentage of participants' intergenerational decisions and the distribution of the intragenerational allocation are represented in Supplementary Table S9 and Supplementary Figure S2, respectively.

Supplementary Table S5. The questionnaires used in our study

Questionnaires	Cronbach's alpha	
	Study 1	Study 2
General trust (GT)(Yamagishi et al., 2015)	.92	.93
Caution (CA)(Yamagishi and Yamagishi, 1994)[3]	.76	.76
Overlap of self, ingroup, and outgroup scale (OSIO; Schubert and Otten, 2002) (only in Study 1)	-	-
Social value orientation triple dominance measure modified version (SVOTDM; Eek and Gärling, 2006) (only in Study 1)	-	-
Social value orientation slider measure (SVOSL; Murphy et al., 2011) (only in Study 2)	-	-

Note. The bottom three questionnaires are questionnaires where Cronbach's alpha cannot be defined.

[3] We used the modified version of Yamagishi and Yamagishi (1994) caution scale, which was made by the its author's group. This scale contains five items: "To get along well in the world, one need to pay attention to the vicious side of people", "One can never be too careful in one's dealings with others.", "In this society, one does not need to be constantly afraid of being cheated."(reverse), "In this society, one has to be alert or someone is likely to take advantage of you.", and "One can avoid falling into trouble by assuming that all people have a vicious streak."

Supplementary Table S6. The descriptive statistics of the personality questionnaire (GT, CA & OSIO)

Study	Presence of the no-decision condition	Evaluation target	GT	CA	OSIO		
					Self & Ingroup	Self & Outgroup	Ingroup & Outgroup
Study 1	With no-decision condition	DM-evaluate (N = 238)	3.82 (1.18)	4.60 (0.88)	4.27 (2.12)	3.63 (2.08)	3.50 (1.93)
		Decision-evaluate (N = 232)	3.82 (1.22)	4.54 (1.01)	4.38 (2.08)	3.66 (2.10)	3.34 (1.88)
	Without no-decision condition	DM-evaluate (N = 238)	3.93 (1.14)	4.52 (0.88)	4.21 (2.12)	3.63 (2.17)	3.30 (1.90)
		Decision-evaluate (N = 236)	3.99 (1.14)	4.50 (0.93)	4.54 (2.11)	3.65 (2.06)	3.36 (1.84)
Study 2 (N = 293)			3.99 (1.21)	4.61 (0.96)	-	-	-

Note. The values in parentheses are standard deviations.

Supplementary Table S7. The SVO classification by SVOTDM (Social value orientation triple dominance measure modified version) in Study 1

Study	Presence of the no-decision condition	Evaluation target	Prosocial		Proself		Unclassified
			Equal	Joint Outcome	Individualistic	Competitive	
Study 1	With no-decision condition	DM-evaluate (N = 238)	151 (63.45%)	16 (6.72%)	15 (6.30%)	0 (0.00%)	56 (23.53%)
		Decision-evaluate (N = 232)	148 (63.79%)	16 (6.90%)	12 (5.17%)	2 (0.86%)	54 (23.28%)
	Without no-decision condition	DM-evaluate (N = 238)	140 (58.82%)	15 (6.30%)	24 (10.08%)	2 (0.84%)	57 (23.95%)
		Decision-evaluate (N = 236)	141 (59.75%)	14 (5.93%)	20 (8.47%)	2 (0.85%)	59 (25.00%)

Note. The values in parentheses are percentages in each condition

Supplementary Table S8. The SVO classification by SVOSL(Social value orientation slider measure) in Study 2

Study	Prosocial		Proself	
	Equal	Altruistic	Individualistic	Competitive
Study 2	229<br>(78.16%)	3<br>(1.02%)	60<br>(20.48%)	1<br>(0.34%)

Note. The values in parentheses are percentages in each condition

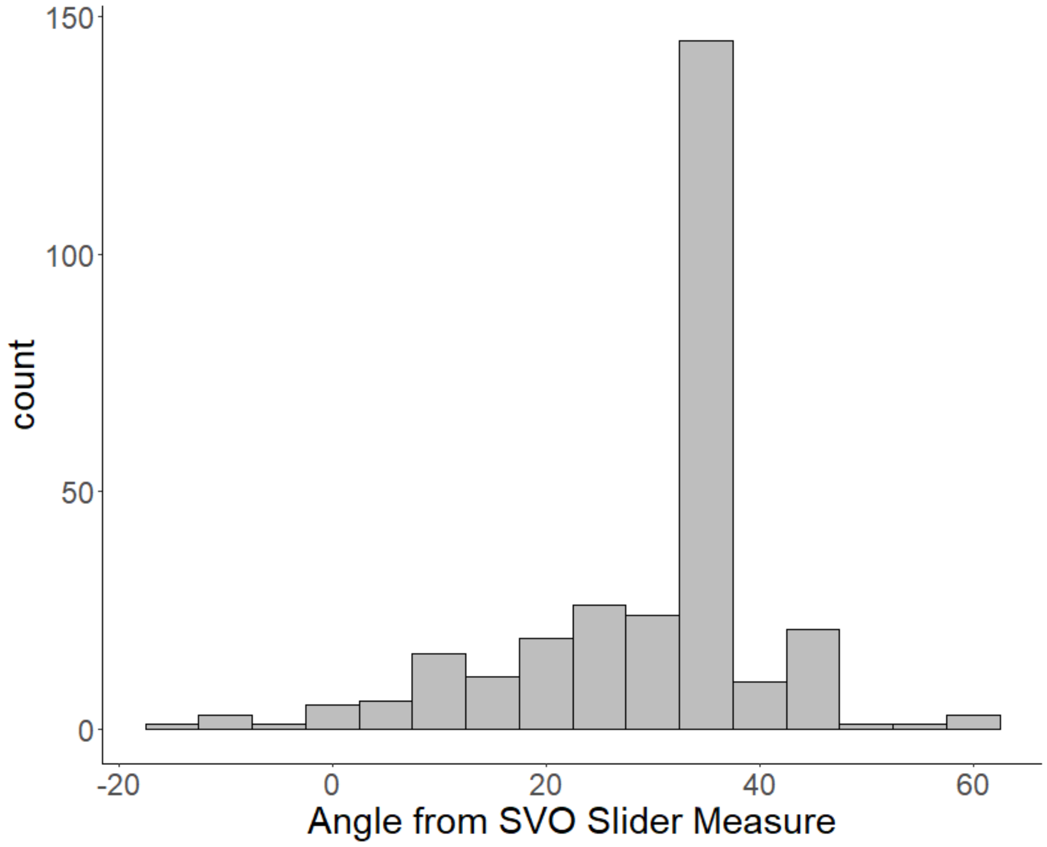

Supplementary Figure S1.

The histogram of Social Value Orientation scores of SVOSL(Social value orientation slider measure) in Study 2.

Note. Please see Murphy et al. (2011) for how to calculate Social Value Orientation score. This histogram was drawn by the ggplot2 package(Wickham, 2016) *for R (ver. 4.0.1)*(R Core Team, 2020).

Supplementary Table S9. The intergenerational decision in ISDG if participants were assigned to the role of DM

Study	Presence of the no-decision condition	Evaluation target	Unsustainable	Sustainable
Study 1	With no-decision condition	DM-evaluate (N = 238)	80 (33.61%)	158 (66.39%)
		Decision-evaluate (N = 232)	68 (29.31%)	164 (70.69%)
	Without no-decision condition	DM-evaluate (N = 238)	89 (37.39%)	149 (62.61%)
		Decision-evaluate (N = 236)	77 (32.63%)	159 (67.37%)
Study 2 (N = 293)			74 (25.26%)	219 (74.74%)

Note. The values in parentheses are percentages in each condition.

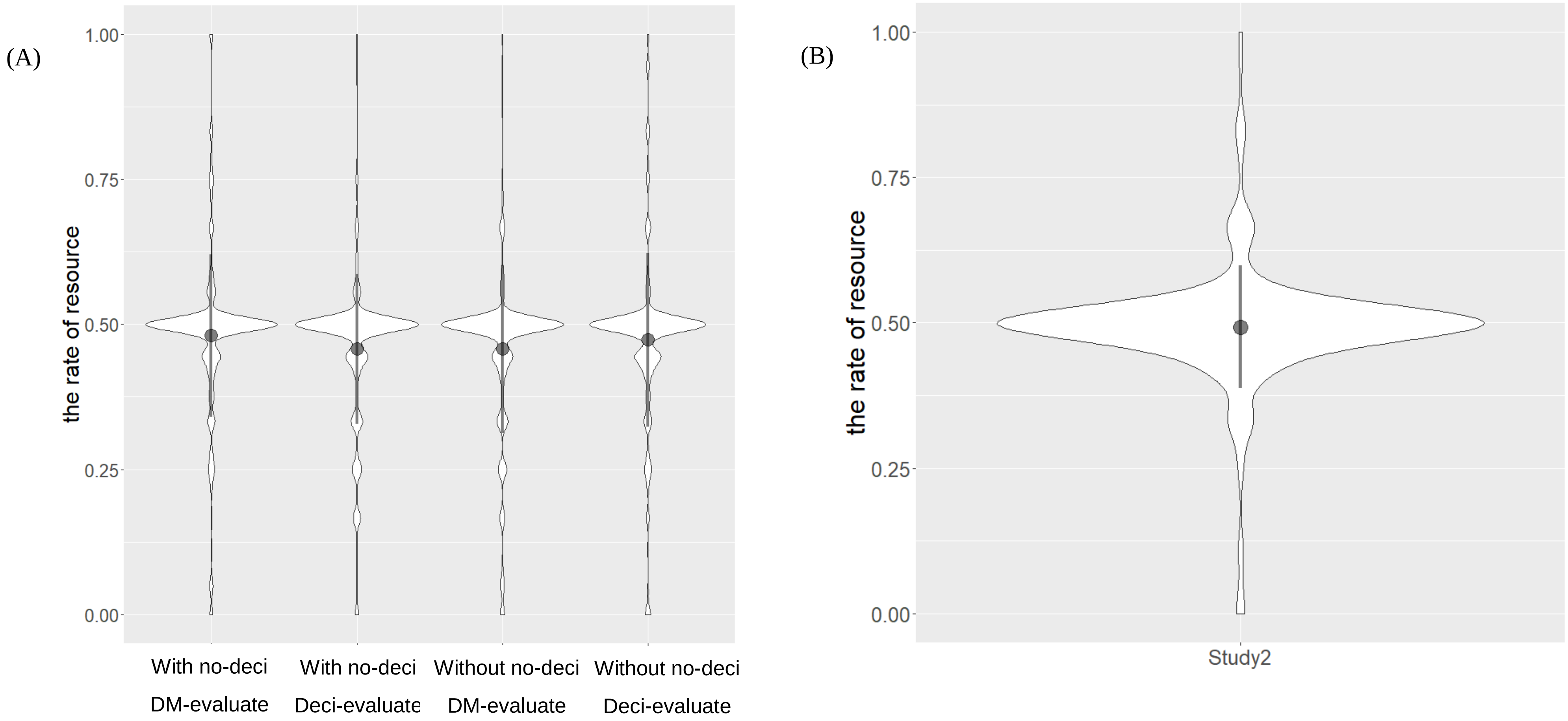

Supplementary Figure S2.
The violin plot of the rate of resource that participants would give to the partner in the intragenerational allocation of ISDG if participants were the DM

Note. *(A) is the allocation in each condition of Study 1 and (B) is the allocation of Study 2. These figures were drawn by the ggplot2 package*(Wickham, 2016) *for R (ver. 4.0.1)*(R Core Team, 2020). *Gray circles were the average, and gray lines were ±1SD.*

We investigated the relationship between personalities and evaluations of unsustainable/sustainable behavior. According to the result of the ANCOVA test, the interaction between participants' intergenerational decisions and the DM's intergenerational decision was found to be consistently significant (Supplementary Tables S10-S13).

In the no-decision condition in Study 1, the only significant effect related to participants' intergenerational decision was the interaction between the participants' intergenerational decision and the DM's intergenerational decision (Supplementary Table S10). According to the simple main effect analysis, the main effect of the DM's intergenerational decision was significant in both the participants who would choose the unsustainable option ($F(1, 466) = 8.645$, Holm-corrected $p = .012$, $\eta_G^2 = .029$) and those who would choose the sustainable option ($F(1, 466) = 67.514$, Holm-corrected $p < .01$, $\eta_G^2 = .095$), but the DM whom participants evaluated more positive was the opposite; participants more positively evaluated the option they prefer as a DM than the other option (Supplementary Figure S3).

In the ANCOVA in the other three intragenerational allocation conditions of Study 1, the interaction between the DM's intergenerational decision and the participants' intergenerational decision was significant (Supplementary Table S11). According to the simple main effect analysis, the main effect of the DM's intergenerational choice was significant in both of participants who would choose unsustainable option A ($F(1, 940) = 17.080$, Holm-corrected $p < .01$, $\eta_G^2 = .010$) and those who would choose sustainable option B ($F(1, 940) = 30.299$, Holm-corrected $p < .01$, $\eta_G^2 = .009$). Again, participants more positively evaluated the option they prefer as a DM than the other option (Supplementary Figure S4). In addition, the second-order interaction between the DM's intergenerational decision, intragenerational allocation, and participants' intergenerational decision was significant. In both of the participants who would choose the unsustainable option A and who would choose the sustainable option B, the effect of DM's intergenerational decision was the

strongest when the intragenerational allocation was equal (the simple effect of interaction between the DM's intergenerational decision and intragenerational allocation; unsustainable option $F(2, 1880) = 5.067$, Holm-corrected $p = .014$, $\eta_G^2 = .002$; sustainable option: $F(2, 1880) = 3.889$, $p = .022$, $\eta_G^2 = .001$).

In Study 2, only the interaction between the DM's intergenerational decision and participants' intergenerational decision was significant among the effects relevant to participants' intergenerational decision (Supplementary Table S12, Supplementary Figure S5). Participants who would choose the sustainable option B evaluated more positively the DM who chose the sustainable option than the DM who chose the unsustainable option ($F(1, 291) = 35.551$, Holm-corrected $p < .01$, $\eta_G^2 = .020$ in the simple main effect analysis). However, the evaluation was not significantly different depending on the DM's intergenerational choice in the participants who would choose unsustainable option A ($F(1, 291) = 0.048$, Holm-corrected $p = .83$, $\eta_G^2 = .000$ in the simple main effect analysis). Although this result seemed to be different from Study 1, this pattern was similar to the evaluation of DM's impression in Study 1[4]. In summary, participants who prefer sustainable options tend to positively evaluate future altruism. This tendency was weak, or even contrary, in participants who prefer unsustainable options.

Finally, we calculated the correlations between other personality indexes (participants' intragenerational allocation, personalities measured by questionnaires) and evaluation in the no-decision condition (Supplementary Table S13). We also indicated the average evaluation for each SVO category in Study 1 (Supplementary Table S14). Note that we have to be careful with the biased distribution of SVO preference, especially the rarity of the proselfs in Study 1.

---

[4] In the Study 2, all participants evaluated the DM's impression. Therefore, this is a plausible result.

Supplementary Table S10.

The ANCOVA with the independent variable of participants' intergenerational choice in ISDG in the no-decision condition of Study 1

Variables	SS	MS	MSe	η_G^2	*F*	df1	df2	*p*
DM's intergenerational decision (InterD)	11.136	11.136	2.376	.005	4.687	1	466	.031*
Evaluation target (Target)	64.876	64.876	1.962	.031	33.068	1	464	.000**
Participants' intergenerational decision (PInterD)	0.254	0.254	1.962	.000	0.130	1	464	.719
InterD × Target	24.553	24.553	2.376	.012	10.333	1	466	.001**
InterD × PInterD	117.669	117.669	2.376	.055	49.520	1	466	.000**
Target × PInterD	0.109	0.109	1.962	.000	0.056	1	464	.813
InterD × Target × PInterD	0.265	0.265	2.376	.000	0.111	1	466	.739
Age	7.668	7.668	1.962	.004	3.909	1	464	.049*
Sex	1.204	1.204	1.962	.001	0.614	1	464	.434

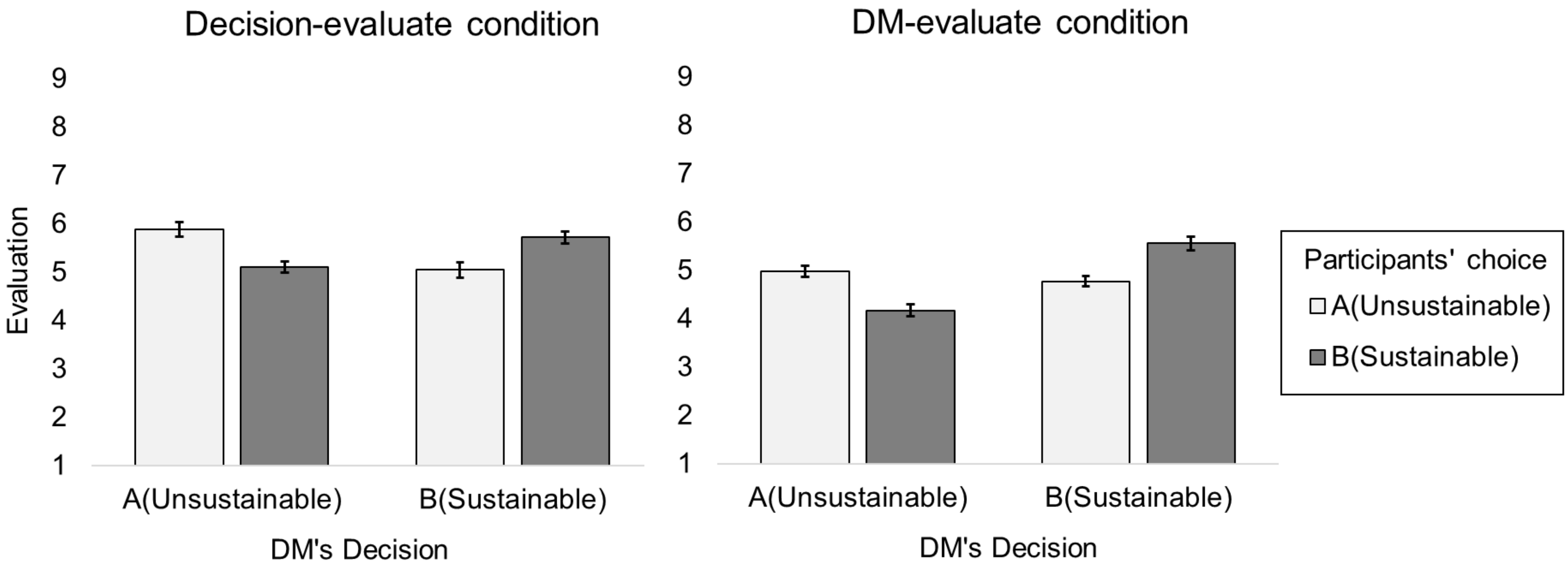

Supplementary Figure S3.

The evaluation of each option by the participants’ intergenerational choice in the no-decision condition of Study 1

Note. Error bars represented standard errors.

Supplementary Table S11.

The ANCOVA with the independent variable of participants' intergenerational choice in ISDG in the three intragenerational allocation condition (selfish, equal, altruistic) in Study 1

Variables	SS	MS	MSe	η_G^2	*F*	df1	df2	*p*
DM's intergenerational decision (InterD)	0.088	0.088	2.000	.000	0.044	1	940	.834
DM's intragenerational allocation (IntraA)	824.645	412.323	2.298	.063	179.411	2	1880	.000**
Evaluation target (Target)	65.303	65.303	5.024	.005	12.998	1	937	.000**
Participants' intergenerational decision (PInterD)	1.425	1.425	5.024	.000	0.284	1	937	.594
InterD × IntraA	2.368	1.184	0.711	.000	1.665	2	1880	.191
InterD × Target	9.097	9.097	2.000	.001	4.549	1	940	.033*
InterD × PInterD	85.747	85.747	2.000	.007	42.879	1	940	.000**
IntraA × Target	48.442	24.221	2.298	.004	10.539	2	1880	.000**
IntraA ×PInterD	13.167	6.583	2.298	.001	2.865	2	1880	.070 †

Variables	SS	MS	MSe	η_G^2	*F*	df1	df2	*p*
Target ×PInterD	2.534	2.534	5.024	.000	0.504	1	937	.478
InterD × IntraA × Target	0.105	0.053	0.711	.000	0.074	2	1880	.923
InterD × Target ×PInterD	5.352	5.352	2.000	.000	2.676	1	940	.102
InterD × IntraA ×PInterD	10.933	5.466	0.711	.001	7.688	2	1880	.001**
IntraA × Target ×PInterD	7.517	3.759	2.298	.001	1.635	2	1880	.200
InterD × IntraA × Target ×PInterD	0.860	0.430	0.711	.000	0.605	2	1880	.540
Age	11.858	11.858	5.024	.001	2.360	1	937	.125
Sex	2.739	2.739	5.024	.000	0.545	1	937	.460
Presence of the no-decision condition	1.045	1.045	5.024	.000	0.208	1	937	.648

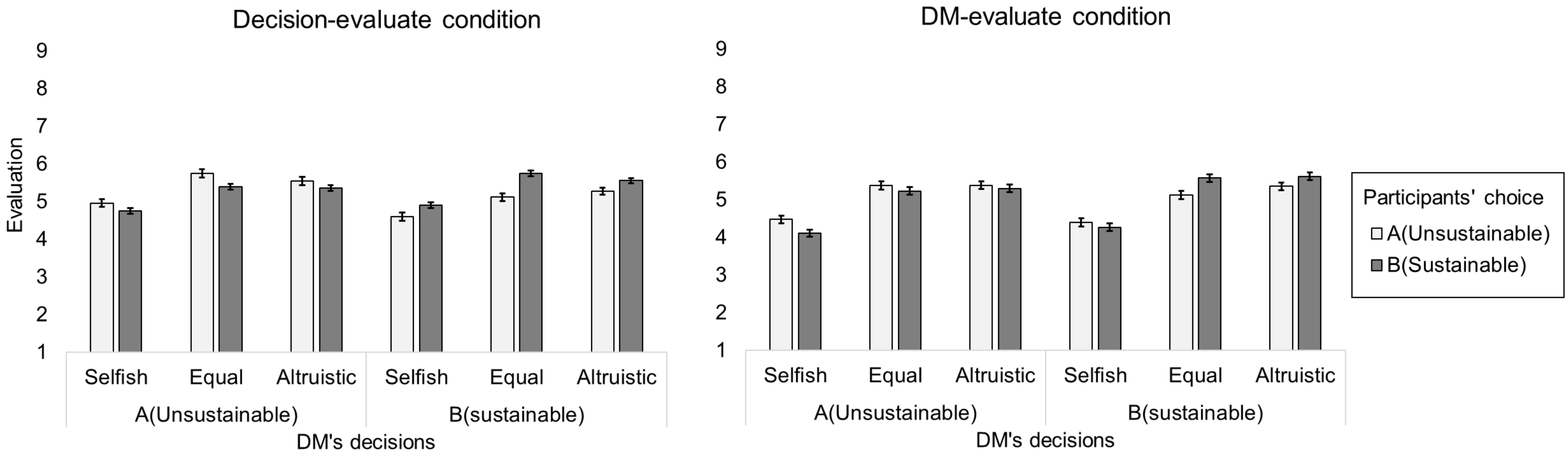

Supplementary Figure S4.

The evaluation of each option by the participants' intergenerational choice in the three intragenerational allocation condition of Study 1

Note. Error bars represented standard errors.

Supplementary Table S12.

The ANCOVA with the independent variable of participants' intergenerational decision in the ISDG in Study 2

Variables	SS	MS	MSe	η_G^2	*F*	df1	df2	*p*
DM's intergenerational decision (InterD)	19.615	19.615	1.932	.004	10.152	1	291	.002**
DM's intragenerational allocation (IntraA)	1079.962	539.981	3.451	.175	156.483	2	582	.000**
Participants' intergenerational decision (PInterD)	0.205	0.205	6.868	.000	0.030	1	287	.863
InterD × IntraA	34.711	17.355	0.951	.007	18.251	2	582	.000**
InterD × PInterD	15.220	15.220	1.932	.003	7.878	1	291	.005**
IntraA ×PInterD	14.534	7.267	3.451	.003	2.106	2	582	.141
InterD × IntraA ×PInterD	3.315	1.658	0.951	.001	1.743	2	582	.177
Age	0.055	0.055	6.868	.000	0.008	1	287	.929
Sex	0.051	0.051	6.868	.000	0.007	1	287	.931
Parenthood	0.013	0.013	6.868	.000	0.002	1	287	.965
Grandparenthood	3.164	3.164	6.868	.001	0.461	1	287	.498

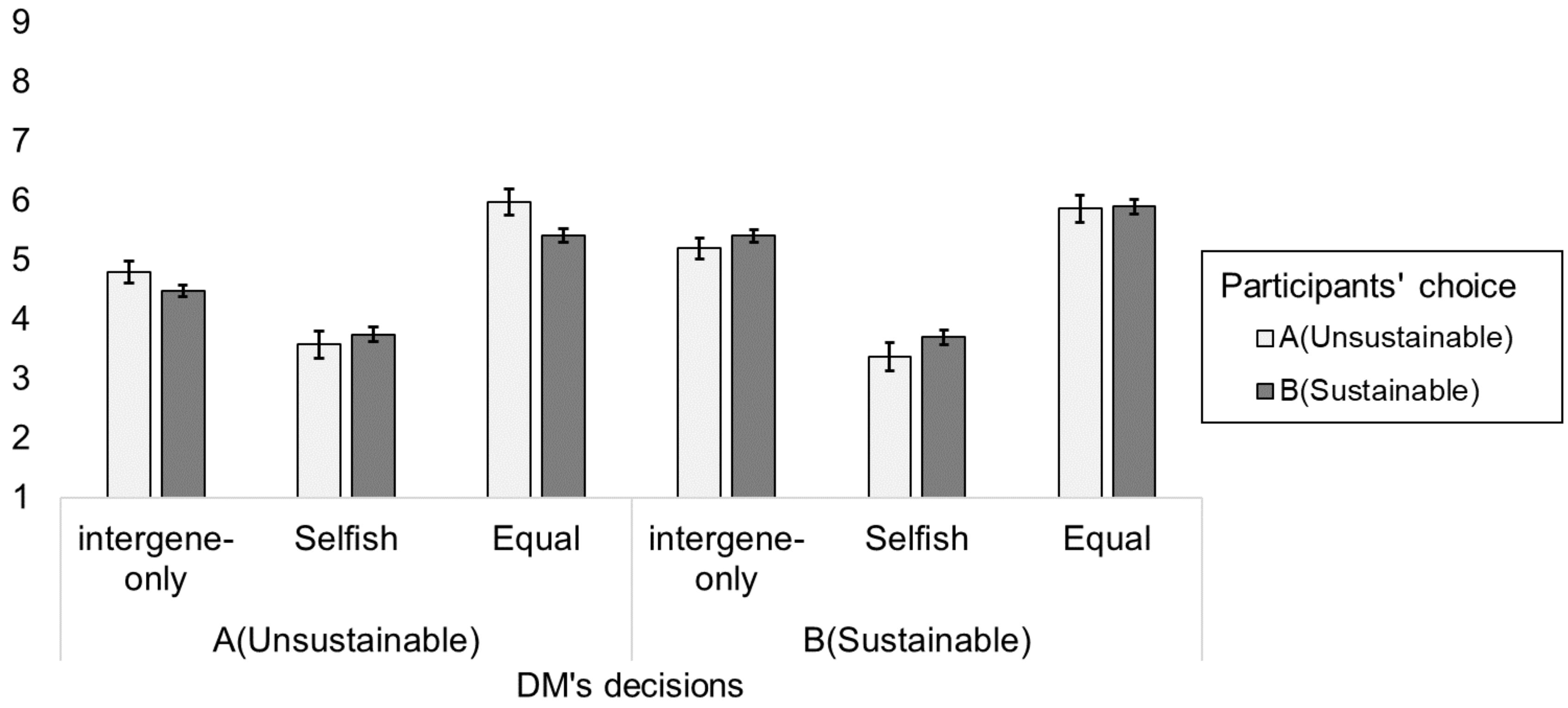

Supplementary Figure S5.

The evaluation of each option by the participants' intergenerational choice in Study 2

Note. Error bars represented standard errors.

Supplementary Table S13. The correlation between personalities and evaluation in the no-decision condition

	A (Unsustainable)			B (Sustainable)		
	Study 1: Decision-evaluate condition	Study 1: DM-evaluate condition	Study 2	Study 1: Decision-evaluate condition	Study 1: DM-evaluate condition	Study 2
Participants' intragenerational allocation toward the member	.02	-.03	-.03	.17*	.14*	.01
Gtrust	-.02	.13*	.35***	.08	.25***	.38***
Caution	.07	.07	.16**	.07	.15*	.16**
OSIO: Distance of self and ingroup	-.08	-.07	-	.00	-.00	-
OSIO: Distance of self and outgroup	-.19**	-.13*	-	.00	-.08	-
OSIO: Distance of ingroup and outgroup	-.10	.01	-	-.03	-.01	-
SVO Slider measure	-	-	-.06	-	-	.10

Note. $*p < .05$; $**p < .01$; $***p < .001$

Supplementary Table S14. The evaluation in the no-decision condition by each SVO in Study 1

SVO	Evaluation target	A (Unsustainable)	B (Sustainable)
Prosocial	Decision-evaluate condition (N = 164)	5.34 (1.53)	5.73 (1.62)
	DM-evaluate condition (N = 167)	4.37 (1.54)	5.61 (1.68)
Proself	Decision-evaluate condition (N = 14)	6.59 (1.72)	4.97 (1.96)
	DM-evaluate condition (N = 15)	4.77 (1.38)	4.45 (1.12)
Unclassified	Decision-evaluate condition (N = 54)	4.98 (0.43)	5.03 (0.58)
	DM-evaluate condition (N = 56)	4.59 (1.33)	4.57 (1.37)

References

Eek, D., Gärling, T., 2006. Prosocials prefer equal outcomes to maximizing joint outcomes. Br. J. Soc. Psychol. 45, 321–337. https://doi.org/10.1348/014466605X52290

Kiernan, K., Tao, J., Gibbs, P., 2012. Tips and strategies for mixed modeling with SAS/STAT® procedures. SASSTAT® Softw. Pap. 332, 18.

Murphy, R.O., Ackermann, K.A., Handgraaf, M., 2011. Measuring Social Value Orientation. Judgm. Decis. Mak. 6, 771–781. https://doi.org/10.2139/ssrn.1804189

R Core Team, 2020. R: A language and environment for statistical computing. R Foundation for Statistical Computing, Vienna, Austria.

Schubert, T.W., Otten, S., 2002. Overlap of self, ingroup, and outgroup: Pictorial measures of self-categorization. Self Identity 1, 353–376. https://doi.org/10.1080/152988602760328012

Wickham, H., 2016. ggplot2: Elegant Graphics for Data Analysis. Springer-Verlag, New York.

Yamagishi, T., Akutsu, S., Cho, K., Inoue, Y., Li, Y., Matsumoto, Y., 2015. Two-component model of general trust: Predicting behavioral trust from attitudinal trust. Soc. Cogn. N. Y. 33, 436–458. http://dx.doi.org/101521soco2015335436

Yamagishi, T., Yamagishi, M., 1994. Trust and commitment in the United States and Japan. Motiv. Emot. 18, 129–166. https://doi.org/10.1007/BF02249397
